# Comparison of diagnosis and treatment of MSSA and MRSA osteomyelitis in children: a case–control study of 64 patients

**DOI:** 10.1186/s13018-023-03670-3

**Published:** 2023-03-13

**Authors:** Yuwei Wen, Chunhua Wang, Haiting Jia, Tao Liu, Jiazhi Yu, Mengyuan Zhang

**Affiliations:** 1 Department of Orthopaedics, Beijing Children’s Hospital, National Center for Children’s Health, Capital Medical University, No. 56, Nalishi Road, Beijing, 100045 China; 2 Department of Orthopaedic Trauma Surgery, Children’s Hospital Affiliate to Shandong University, Jinan Children’s Hospital, No. 23976 Jingshi Road, Huaiyin District, Jinan, 250022 Shandong China; 3 Department of Peking Union Medical College, Class of 2025, Beijing, 100730 China

**Keywords:** Osteomyelitis, Methicillin-resistant *Staphylococcus aureus*, Drug resistance, Microbial, Child

## Abstract

**Background:**

We aimed to compare the clinical characteristics of acute osteomyelitis caused by methicillin-resistant *Staphylococcus aureus* (MRSA) and methicillin-sensitive *Staphylococcus aureus* (MSSA) in children.

**Methods:**

We retrospectively analyzed the data of 64 children treated between September 2017 and June 2021. Based on the bacterial culture results, they were divided into MRSA and MSSA infection groups. Both groups were treated with debridement and vacuum-assisted closure for negative pressure drainage. Parameters including clinical manifestations, number of operations, length of hospital stay, inflammatory indicators, and concurrent arthritis were compared between the two groups.

**Results:**

In the MRSA infection group, there was one case each of residual joint stiffness and pathological fracture. Conversely, the MSSA group had two cases of residual joint stiffness. The MRSA infection group was more prone to high fever (*t* = 3.61, *P* = 0.001), white blood cell count elevation (*t* = 2.41, *P* = 0.022), arthritis (*X*^2^ = 7.48, *P* = 0.013), metastatic abscess (*X*^2^ = 4.78, *P* = 0.042), and a shorter length of progression from onset to admission (*t* = − 2.04, *P* = 0.046); however, it required more surgeries (*t* = 2.68, *P* = 0.009) and longer hospital stay (*t* = 2.04, *P* = 0.045).

**Conclusions:**

Pediatric acute osteomyelitis caused by MRSA is more prone to cause high fever and markedly elevated of white blood cell count, and is often accompanied with suppurative infection of adjacent joints and metastatic abscesses, thus requiring more surgeries and longer hospital stay.

## Introduction

Acute hematogenic osteomyelitis (AHO), typically occurring in the distal femur and proximal tibia, is one of the most common infectious diseases in children. It is usually caused by a blood-borne infection [1]. However, few cases are caused by the spread of an adjacent soft tissue infection or secondary open fracture. Owing to the different development processes of bone and epiphyseal vessels in children, the incidence of AHO varies by age and sex. The incidence is approximately 1:5000–1:10,000, and it is twice as common in boys than in girls [2]. AHO has a sudden onset and rapid progression and may cause serious complications or even endanger the lives of affected children if not treated in time [3]. Although the incidence may vary across groups, the microbial epidemiology is basically consistent, in which methicillin-sensitive *Staphylococcus aureus* (MSSA) plays a major role [4]. However, as MSSA virulence and aggressiveness have evolved, the pathophysiology and epidemiology of infection have changed. In recent decades, the incidence of methicillin-resistant *Staphylococcus aureus* (MRSA) infection in children has increased. MRSA is more virulent and invasive than MSSA, causing complex and deep tissue infections, including skeletal muscle infections, which require more comprehensive evaluation and treatment [5]. Osteomyelitis caused by MRSA infection is more likely to be cause high fever, tachycardia, pain, and claudication than that caused by MSSA infection [6]. In this study, the clinical manifestations, inflammatory indicators, and therapeutic effects of acute osteomyelitis caused by MRSA and MSSA infections were compared to further analyze the differences and guide clinical treatment.

## Materials and methods

This was a retrospective case–control study. It included 64 patients with acute osteomyelitis confirmed by imaging examination and pathology between September 2017 and June 2021 at Jinan Children’s Hospital.

*Inclusion criteria* 1**.** Acute osteomyelitis confirmed by imaging examination and pathology. 2. Bacterial culture yielded MRSA or MSSA. 3. The patient was treated with debridement and vacuum-assisted closure (VAC) for negative pressure drainage. 4. Age < 18 years.

*Exclusion criteria* 1. Chronic or subacute osteomyelitis. 2. Immunodeficiency 3. Concurrent hematological diseases.

**Fig. 1:**
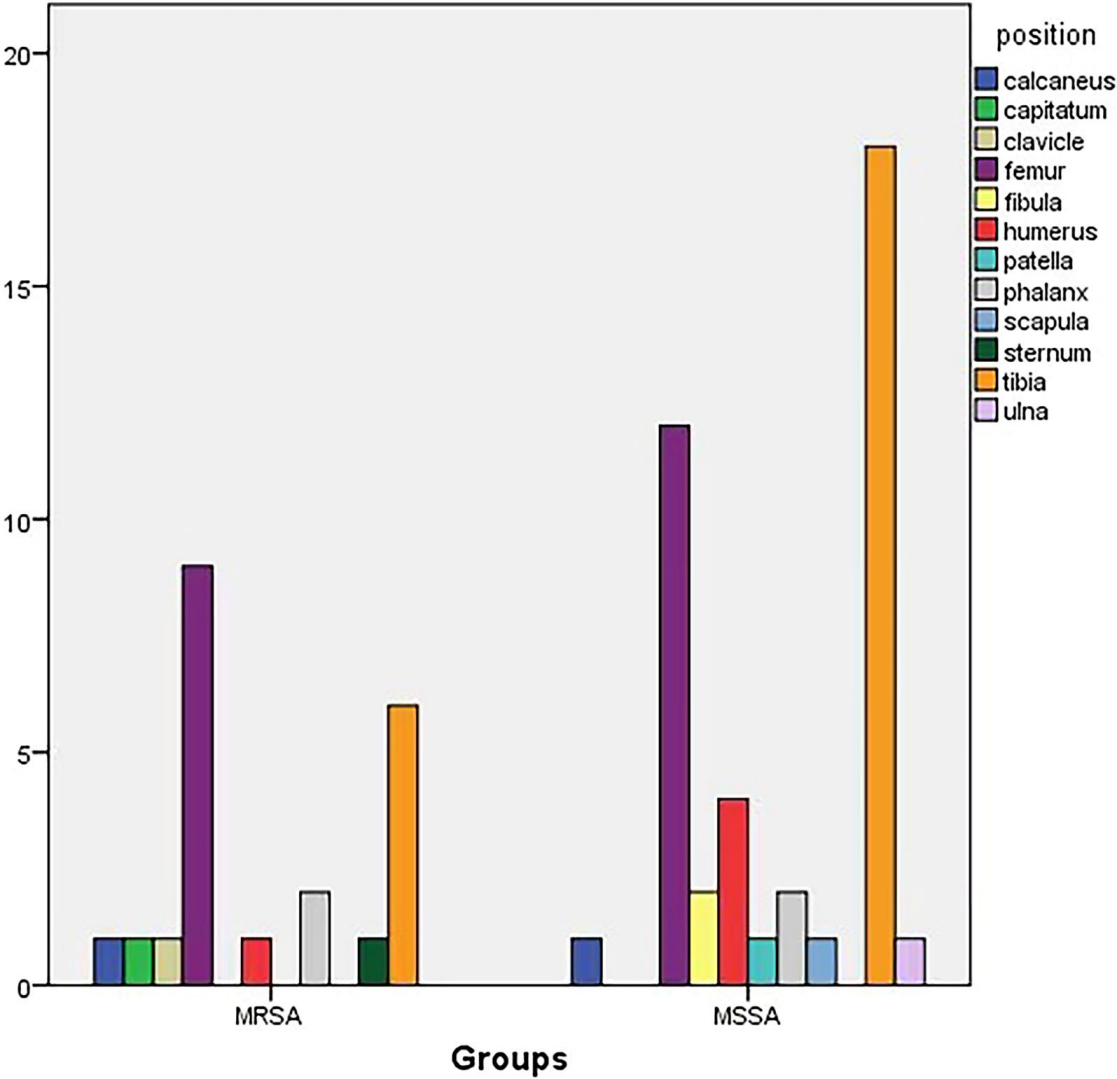
Distribution of affected sites between the MRSA infection group (22 patients) and MSSA infection group (42 patients)

This study included 37 boys and 27 girls. The age ranged from 19 days to 12 years, with an average age of 60 months. Most of the patients had no obvious causes of the disease at the onset, and the affected sites were mainly the femur and tibia (Fig. 1). The clinical manifestations were fever, local redness, tenderness, and limited limb movement. Patients with arthritis had corresponding joint swelling, effusion, and limited movement. Laboratory investigations such as routine blood examinations, c-reactive protein (CRP), erythrocyte sedimentation rate (ESR), and imaging examinations such as MRI were performed on all the patients. Based on the results of the bacterial culture, they were divided into MRSA and MSSA infection groups with 22 and 42 patients, respectively. Our study was approved by the ethical committee of Jinan Children’s Hospital (Ethical code: QLET-IRB/T-2021020).

### Treatment

**Fig. 2:**
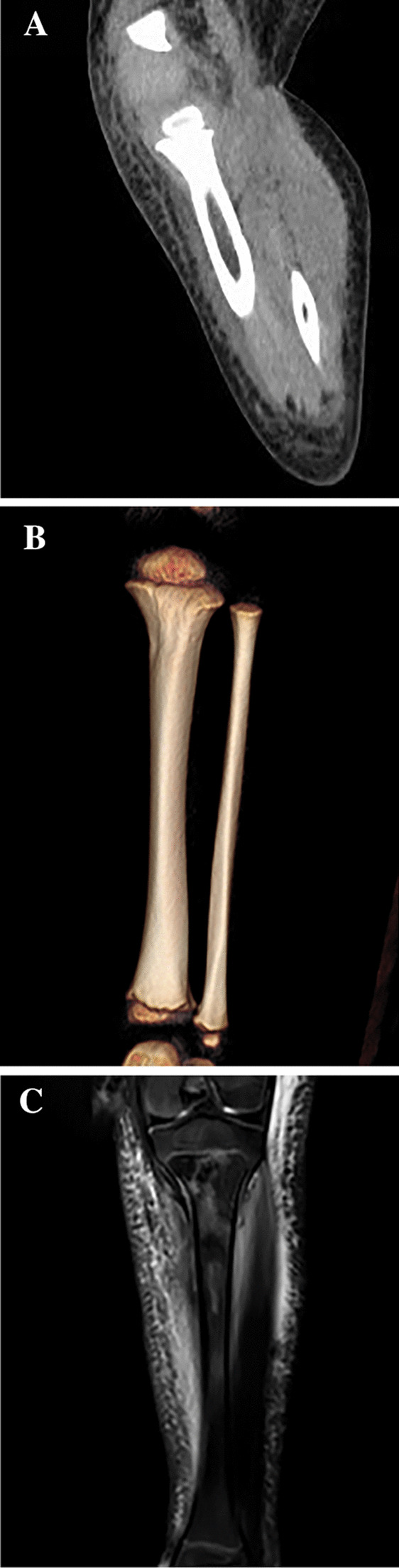

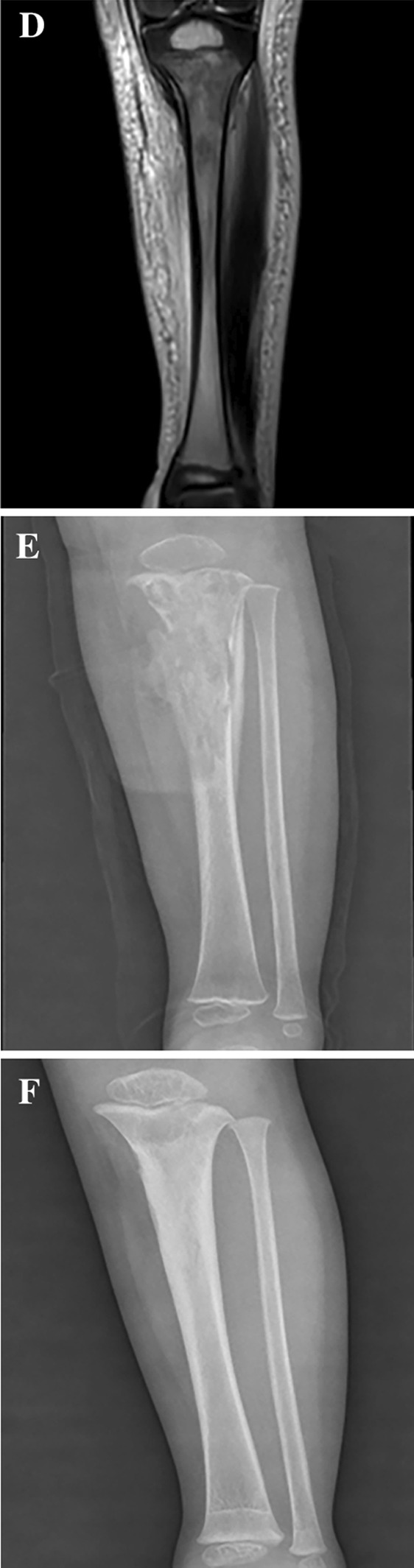
**A**, **B** A 2-year-old boy presented with high fever, swelling, and tenderness of the left lower leg. The inflammatory indexes on admission were all elevated. Computed tomography revealed soft tissue swelling of the left lower leg but no fracture or bone destruction. An MRI examination revealed abnormal signals in the proximal tibia soft tissue and bone marrow cavity (**C**, **D**). The treatment administered was an antibiotic combination with tibial cortex fenestration, lesion removal, VAC negative pressure drainage; postoperative pathological findings suggested suppurative osteomyelitis. Bacterial culture suggested MRSA, following which the antibiotic was replaced with vancomycin. The VAC negative pressure was removed after four rounds of surgical treatment, and the total duration of hospital stay was 60 days. The inflammatory indicators and body temperature were normal at discharge, and the re-examination radiograph indicated bone destruction at the proximal tibia (**E**, **F**). Nine months after surgery, radiographs indicated remodeling of the proximal tibia (**G**), with normal lower limb function and no postoperative complications

After admission, all patients were administered first or second-generation cephalosporin antibiotics and treated with bone cortex fenestration, lesion removal, and VAC negative pressure drainage. Intraoperatively, the drainage fluid of the medullary cavity and the surrounding pus were collected for drug sensitivity culture, while the affected bone was collected for pathological examination. The negative pressure dressing device was wrapped with oil gauze and then filled to the surface of the bone cortex fenestra. The machine was connected to continue negative pressure suction, and the pressure used was generally between 50 and 100 mmHg based on the patient’s age and lesion location. For patients with suppurative arthritis diagnosed by preoperative imaging examination, joint debridement was performed at the same time. Intravenous antibiotics were administered pre and postoperatively; they were continued for 3 weeks followed by oral antibiotics for 3 weeks. First- or second-generation cephalosporins were routinely used preoperatively. Antibiotics were selected postoperatively based on the results of the drug sensitivity test. If bacterial culture revealed MSSA, the preoperative antibiotic was continued; if it revealed MRSA, the antibiotic was replaced with vancomycin or an antibiotic with corresponding sensitivity. One week postoperatively, the decision to remove negative pressure or perform surgical procedures again was made based on the assessment of the specific condition of the patient. Typical cases are shown in Fig. 2.

### Statistical methods

Statistical analysis was performed using standardized statistical software (Statistical Package for Social Science, version 23.0; SPSS, Inc., Chicago, IL). The Shapiro–Wilk test was used to verify the normal distribution of measurement data. Normally distributed variables were expressed as mean and standard deviation, while non-normally distributed data were expressed as median (25th, 75th interquartile range); data were analyzed using the t test and Wilcoxon test. Enumeration data were expressed as frequency, rate, or composition ratio and tested using the Chi-square test. *P* values of < 0.05 were considered statistically significant.

## Results

In the MRSA infection group, one patient had residual joint stiffness and one had a pathological fracture. Two patients in the MSSA group had residual joint stiffness. There were no cases of septic shock. The patients who had compensatory shock may have been missed during diagnosis owing to the absence of obvious clinical symptoms. As there were few postoperative complications in the two groups, no statistical analysis was performed to assess this.

Statistical analysis showed that the mean age was 60.17 ± 40.11 months in the MRSA group and 41.90 ± 42.96 months in the MSSA group, indicating no significant difference between the two groups. There were no significant differences in sex and fever ratio (*X*^2^ = 3.45, *P* = 0.080) as well. However, the fever degree in the MRSA group was higher than that in the MSSA group, and the difference was statistically significant. Additionally, the MRSA group was more likely to develop suppurative arthritis and metastatic abscesses than the MSSA group. The time from onset to hospital visit was shorter in the MRSA infection group. There were no significant differences in CRP and ESR between the two groups, but white blood cell (WBC) count in the MRSA group increased more significantly than in the MSSA group. Table 1 summarizes the clinical data of the two groups.

**Table 1:** Statistical analysis results of the MRSA group and MSSA group

Group	MRSA	MSSA	*t*/*x*^2^	*P*
Items				
Age(m)	60.17 ± 40.11	41.90 ± 42.96	0.71	0.479
*Sex*				
Male	12	25	0.15	0.792
Female	10	17		
Visit time(d)	6.00 ± 2.51	9.40 ± 10.23	− 2.044	0.046
Length of stay(d)	36.59 ± 13.53	30.64 ± 9.55	2.04	0.045
*Fever*				
Y	16	38	3.45	0.080
N	6	4		
Temperature(℃)	39.44 ± 0.82	38.72 ± 0.596	3.61	0.001
Number of operations	3.41 ± 1.40	2.69 ± 0.75	2.68	0.009
*Arthritis*				
Y	10	6	7.48	0.013
N	12	36		
*Metastatic abscess*				
Y	5	2	4.78	0.042
N	17	40		
WBC(× 10^9^/L)	15.79 ± 7.29	11.57 ± 5.23	2.41	0.022
CRP(mg/L)	65.68 ± 55.23	57.28 ± 68.13	0.498	0.620
ESR(mm/h)	52.27 ± 23.13	62.88 ± 32.41	− 1.36	0.178

## Discussion

The most common pathogenic bacteria associated with AHO in children are MSSA and MRSA [7]. Recent studies have demonstrated that MRSA has stronger virulence and invasiveness and has become an increasingly important cause of AHO [6]. Our study indicated that MRSA infection results in the need for more surgeries and prolong hospital stay because of high fever, and markedly elevated WBC count, accompanied with suppurative infection of adjacent joints and metastatic abscesses.

Clinically, first- or second-generation cephalosporins are routinely used to treat acute osteomyelitis in children. However, they do not yield satisfactory results for children with MRSA infections. The selection of antibiotics for children with MRSA infections varies depending on the bacterial strain and lesion location. Biologically, MRSA can be divided into community-associated MRSA (CA-MRSA) and hospital-associated MRSA (HA-MRSA). The clinical diagnostic criteria for CA-MRSA recommended by the U.S. Centers for Disease Control and Prevention are [8]: (1) MRSA strains isolated from patients in the outpatient department or within 48 h of admission; (2) The patient has no indwelling catheter or artificial medical device; and (3) No history of hospitalization or contact with nurseries, nursing homes, or asylum institutions within 1 year. The internationally recognized molecular biological standard for distinguishing CA-MRSA from HA-MRSA is that CA-MRSA carries CCmec IV–VI, while HA-MRSA carries SCCmec I–III [9, 10]. In Iran, the United States, Europe, and most Asian countries, the distribution of SCCmec types indicated that SCCmec type III was the most prevalent, which emphasizes the nosocomial origin of these strains [11]. A remarkable result in the study by Navidinia et al. was the high percentage of MRSA infections among health care providers [10]. Unfortunately, the incidence rate of MRSA in health care provider carriers is lower than that shown in clinical samples. Therefore, MRSA screening is not often performed for these individuals.

The children with MRSA infections admitted to our hospital did not undergo strain identification using molecular biology, but based on the clinical diagnostic criteria, they were infected with the CA-MRSA strain. Based on the research on MRSA resistance in China, CA-MRSA has high resistance to most of the current antibacterial drugs; however, no strain is resistant to linezolid and vancomycin [12]. Therefore, we administered vancomycin or linezolid for the patients with MRSA infections.

Distinguishing MRSA from MSSA is difficult in a clinical setting before the results of bacterial cultures are obtained. However, early identification of MRSA and MSSA is crucial to timely administer sensitive antibiotics and avoid the emergence of more complex inflammation and spread of infection. Temperature > 38 °C, hematocrit < 34%, white blood cell count > 12,000 cells/ml and CRP > 13 mg/L are considered the four clinical predictors of MRSA infection. The positive prediction rate of all four factors is 92%; three factors is 45%; two factors is 10%; one factor is 1%; and no factors is 0% [6]. In our study, patients infected with MRSA had received medical treatment earlier. Additionally, patients infected with MRSA were more likely to develop high fever, with more obvious WBC count elevation; however, there was no significant difference in terms of CRP and ESR as compared to the MSSA group. Based on our experience, we suggest early administration of vancomycin in anti-infection treatment for children with high fever and WBC count twice the normal value to avoid delay in using sensitive antibiotics when waiting for the results of bacterial culture. This strategy may prevent more complicated infections or other complications.

No significant differences in radiographic findings have been reported between acute osteomyelitis with MRSA and MSSA infections. Early bone changes usually do not appear until 10 to 12 days later. Generally, only when at least 30% of the bone is destroyed are significant changes visible on conventional radiography [13]. Cortical erosion usually takes 2–3 weeks to develop, and there may be significant bone destruction in the advanced stage of the disease [1]. MRI is crucial in the early diagnosis of osteomyelitis. It is highly sensitive and specific, with reported values of 88–100% and 75–100%, respectively [14]. In addition to determining the affected site, MRI plays a key role in determining soft tissue involvement, delineating bone and soft tissue abscesses, and indicating co-existing joint lesions (such as effusion or synovitis) [15]. It is also valuable for determining the anatomical and spatial scope of infection, which can guide clinical surgical decisions and choice of surgical methods [5]. Therefore, all our patients in this study underwent MRI examinations, and the surgical method and window size of bone cortex were all subsequently determined based on the results of MRI. Owing to the fact that postoperative changes take relatively longer to be reflected on MRI, radiographs combined with data on inflammatory indicators and local symptoms can be employed to understand local bone destruction and recovery.

Although the surgical indications, specific guidelines, and specific surgical techniques or scope of AHO have not been extensively studied and clearly defined [16], surgical intervention and drainage have been reported as a necessity to avoid disease progression [17]. Additionally, surgery alters the process of bone necrosis, which reduces the vasculature and therefore the penetration of the antibiotic at the site of infection; it removes the demineralized bone, and cleans the surrounding soft tissue, thereby reducing the bacterial load [18]. We use VAC for negative pressure drainage. VAC can create a closed and moist wound healing environment, stimulate cell reproduction, increase local blood flow, promote granulation tissue growth, and accelerate wound healing [19, 20]. One week postoperatively, negative pressure drainage was performed again or removed depending on the body temperature, inflammatory indicators, and results of the pus culture. The criteria for removing VAC negative pressure were: no bacterial growth in pus culture and satisfactory control of temperature and inflammatory indicators. This study demonstrated that the MRSA infection group required more surgeries and longer hospital stay than the MSSA infection group. The analysis for these parameters is performed for the following reasons: (1) The children with MRSA infection had not undergone drug sensitivity tests preoperatively, the clinical judgment was inaccurate, and sensitive antibiotics could not be administered timely. (2) MRSA has stronger virulence and aggressiveness, and it is prone to being complicated with metastatic abscess and suppurative arthritis, which increases the difficulty of operation and lesion removal. (3) The fenestration of the bone cortex was too small to allow adequate drainage in some patients. Therefore, for patients with a high suspicion of MRSA infection, sensitive antibiotics can be administered early before the results of the drug sensitivity. A preoperative MRI examination was completed to confirm the presence of joint lesions. A detailed physical examination was conducted to determine the presence of metastatic abscesses. Joint lesions or metastatic abscesses can be treated at the time of surgery. Based on the MRI examination, the window opening range should be sufficient during the operation and can be appropriately expanded; otherwise, it would cause poor drainage, poor surgical effect, and unsatisfactory infection control, resulting in more complex infections and serious complications.

Long-term complications of pediatric osteomyelitis include loss of limb function and growth disorder [21], and complications are more common in children with MRSA infection [22]. Among the 64 patients, two experienced complications in each group. Because of limited data, statistical analysis was not performed. In the MRSA infection group, one patient with osteomyelitis of the femur combined with hip arthritis experienced hip stiffness and was readmitted for surgical release; however, he still had residual hip mobility limitation and an abnormal gait. Another patient with osteomyelitis of the femur had a pathological fracture 1 month after discharge, which did not heal after plaster fixation. About 1 year postoperatively, the patient was readmitted for lesion removal, fracture reduction, and drug-loaded bone cement filling. The fracture healed well with no limitation of lower limb function. Some studies have demonstrated that pathological fracture is related to MRSA expressing USA300-0114 [23]. USA300, as a major international epidemic clone, has been remarkable for the rapidity of its dissemination within the community and hospital. Goudarzi et al. [24] demonstrated that USA300 accounted for 17.2% of the MRSA isolates, which was higher among the hospital-onset cases (75%) than the community-onset cases (25%). However, as our hospital has not conducted molecular studies, further conclusions cannot be drawn. In the MSSA group, two children experienced residual joint stiffness but obtained satisfactory range of motion after rehabilitation exercise.

### Limitations

This is a retrospective study, and future research studies should consider a prospective design to validate the findings. The surgeries were performed by different doctors, and there may have been differences in surgical techniques. The number of cases differed between the two groups, which may have led to statistical error. The overall number of cases is small; therefore, studies with large sample sizes are required.

## Conclusions

Acute suppurative osteomyelitis in children is usually caused by a blood-borne infection, with *Staphylococcus aureus* infection being the most common cause. MRSA accounts for an increasing proportion of AHO cases owing to its stronger virulence and invasiveness. Osteomyelitis caused by MRSA infection is more likely to cause high fever and markedly elevated of WBC count, and is often accompanied by suppurative infection of adjacent joints and metastatic abscesses, necessitating more surgeries and longer hospital stay. To avoid infection spread in children with persistent high fever who have a significant increase in WBCs and high suspicion of MRSA infection, sensitive antibiotics should be administered early, before the results of bacterial cultures are obtained. An MRI examination, cortical fenestration, lesion removal, and VAC negative pressure drainage should be performed as soon as possible. Joint lesions and metastatic abscesses should be treated at the same time to reduce the number of surgeries and complications and obtain satisfactory therapeutic results.

## Data Availability

The datasets used and/or analyzed during the current study are available from the corresponding author on reasonable request
